# Management of a Rare Case of Central Nasal Dermoid Cyst Deformity in an Adult Patient

**DOI:** 10.7759/cureus.49652

**Published:** 2023-11-29

**Authors:** Ali Adil, Ayisha Ayub

**Affiliations:** 1 Plastic and Reconstructive Surgery, Ayesha Bashir Hospital, Gujrat, PAK; 2 Research and Development, Ayesha Bashir Hospital, Gujrat, PAK

**Keywords:** central dermoid, dermoid management, rare case, nasal dermoid, dermoid cyst

## Abstract

Congenital nasal dermoid and sinus cysts (NDSCs) are rare congenital deformities with a prevalence rate of 0.005% to 0.0025%. Early diagnosis is usually made during the first three years of life, but in some cases, the diagnosis may be delayed. The present case study elaborates the treatment course of a 22-year-old adult with a rare congenital nasal midline dermoid cyst. The patient had no family history of the deformity, and intracranial extensions were also ruled out before surgery. Open rhinoplasty technique was used along with osteotomies during the surgical process. The cyst was removed in entirety. The total operating time was six hours, and no complications were observed during the intra-operative or post-operative period. In conclusion, the case presentation focuses on various techniques and methods that can be used during surgery that have not been practiced before to correct the deformity while achieving a good aesthetic result as well.

## Introduction

Congenital nasal dermoid and sinus cysts (NDSCs) are rare deformities that constitute the most common type of inborn nasal midline lesions with an incidence rate of 0.005% to 0.0025% [1]. NDSCs account for 1-3% of all the different types of dermoid cysts and about 11-12% of total head and neck dermoids [2,3]. They also attribute to 61% of all midline nasal lesions in children [4,3]. An NDSC emerges from the ectoderm that appears from neuroectodermal and ectodermal inseparation [5].

The dominant characteristics of NDSCs include the presence of fistula orifice on the nose midline or on the face midline between the eyebrow and nasal columella, in addition to a second fistula in the inner canthus in some cases; the cyst generally presents as a round mass with elasticity at the nose midline, recurrent occurrence of both dermoids and cysts following an infection, and intracranial invasion as observed in 20% of NDSC patients as opposed to some patients who present with a wide orbital space [6].

Diagnosis is established on the basis of the signs and symptoms of the patient and computed tomography (CT) scan and/or magnetic resonance imaging (MRI) examination.

Early diagnosis is usually made during the first three years of life in most of the cases, but in some cases, the diagnosis may be prolonged [4,7,8]. Prolonged diagnosis can lead to complications, such as midline deformities of the nose, repeated infections, obstruction of airway, and intracranial complications [3]. Surgery is the treatment of choice for NDSCs. Since their identification in 1982, many surgical methods have been proposed to treat NDSCs, ranging from the initial invasive nasal incisions to trauma limiting incisions and, finally, to minimally invasive incisions.

The present case study elaborates the treatment course of a 22-year-old adult with a rare congenital nasal midline dermoid cyst focusing on various techniques and methods used during surgery to correct the deformity while achieving a good aesthetic result as well.

## Case presentation

**Figure 1:**
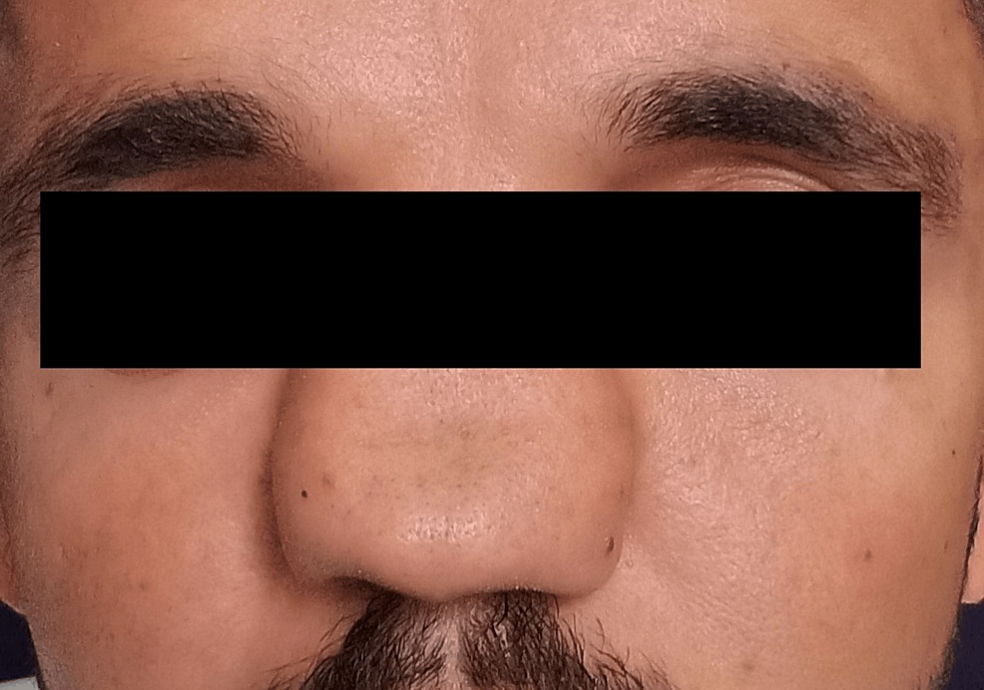
Pre-treatment picture

**Figure 2:**
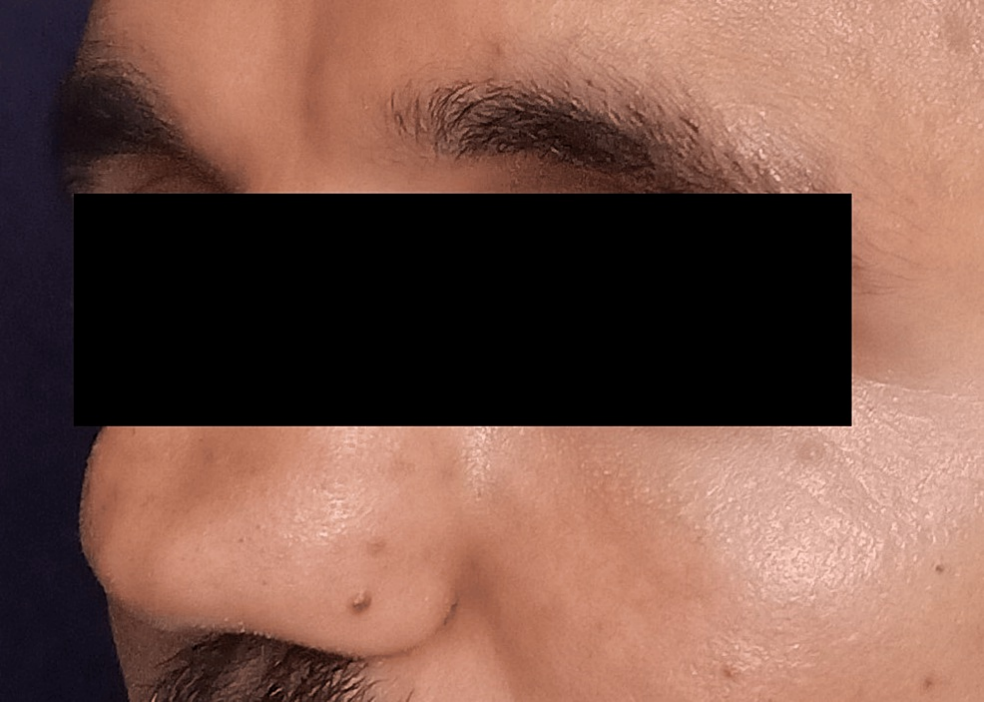
Pre-treatment picture (side view)

**Figure 3:**
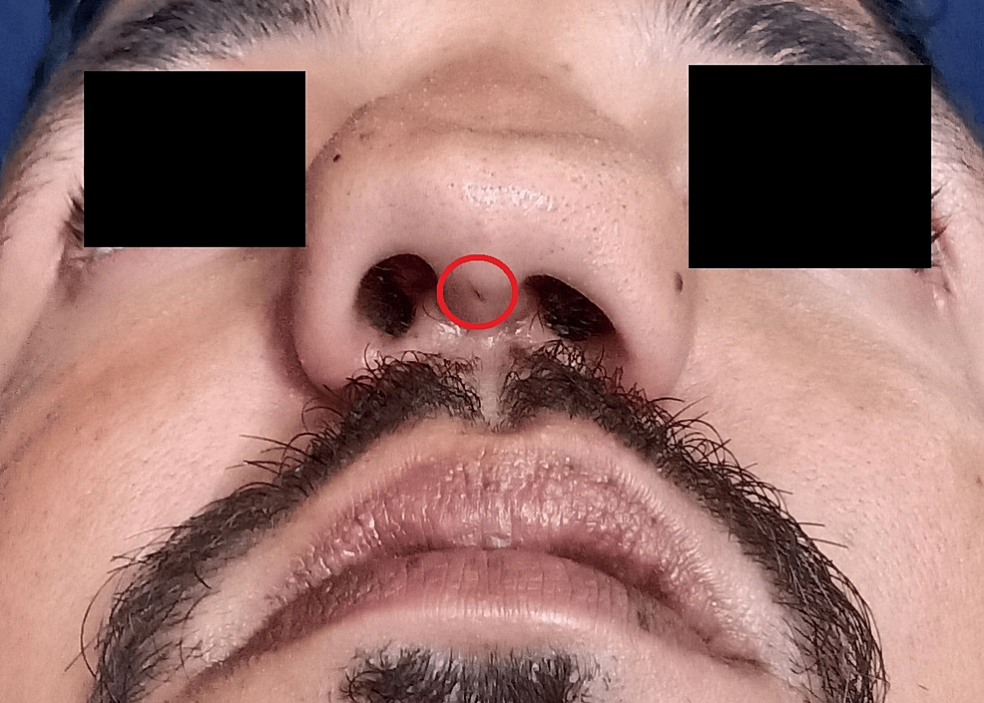
Picture showing a small fistula

A 22-year-old patient presented with painless, slowly growing swelling of the nose from childhood. There was a mild airway problem with nasal breathing. The patient could express a semisolid whitish material from the columellar opening with digital pressure, which resembled a closed comedone. The nasal deformity comprised of a very wide radix, increased intercanthal distance, very broad bony vault, and dorsum. Dorsal aesthetic lines were splayed with ballooning out and depression in the supra-tip area. There was poor dorsum and tip support (saddle nose) and tip projection (Figures 1, 2, 3). There were hard vertical ridges in the bilateral nostrils, suggestive of a bifid caudal septum. Posteriorly, there was bulging of the septum bilaterally. There was webbing of the internal angle. The cottle sign was negative. There was no symptom or sign suggestive of a neurologic abnormality. The CT scan finding included a large space occupying a lesion replacing the catilageous septum, perpendicular plate of the ethmoid and vomar bone. Nasal processes of the maxillary bone and nasal bones severely deformed and ballooned out. The scan confirmed no intracranial extension.

He had no specific history of dermoid cysts within his family and no maxillofacial trauma. The parents also had no consanguineous relation. The patient had received no treatment thus far, so a treatment plan was devised for the surgical removal of the dermoid cyst.

Treatment progress

All necessary tests were performed, and after a detailed clinical evaluation by an anesthetist and surgeon, the patient was declared fit for surgery. The following treatment course was followed:

Rhinoplasty

We performed an open technique rhinoplasty under general anesthesia (GA) with endotracheal tube (ETT) intubation. This was combined with local anesthesia (LA). The LA contained 2% xylocain with 1:100,000 adrenaline and was infiltrated in the incision sites, septum, lining of the deformed lower lateral cartilages (LLCs), dorsum, and osteotomy sites at the start of surgery and was repeated after two hours intraoperatively to reduce bleeding. As a part of our rhinoplasty protocol (well supported by literature and standard practice), LA was infiltrated to the operative site to achieve two main objectives: 1. Lignocain in LA helps reduce the intraoperative pain stimulus, so there is no fluctuation of heart rate and blood pressure, and there is less need of IV analgesia intraoperatively. 2. Adrenaline in LA helps in vasocontriciton of the surgical site, there is less blessing in the area, the surgical field remains clear, and there is less need of electrocautery during the procedure.

**Figure 4:**
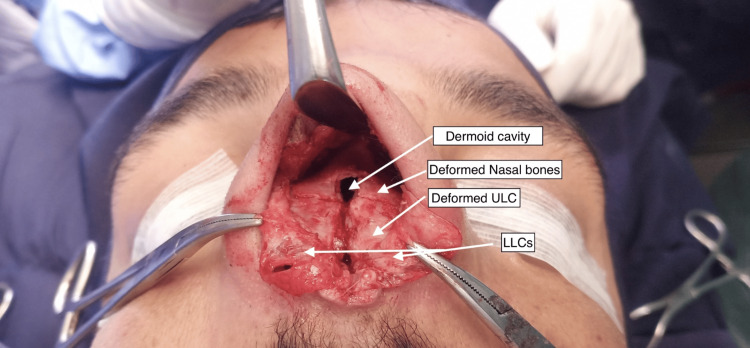
Preoperative picture showing the dermoid cavity

**Figure 5:**
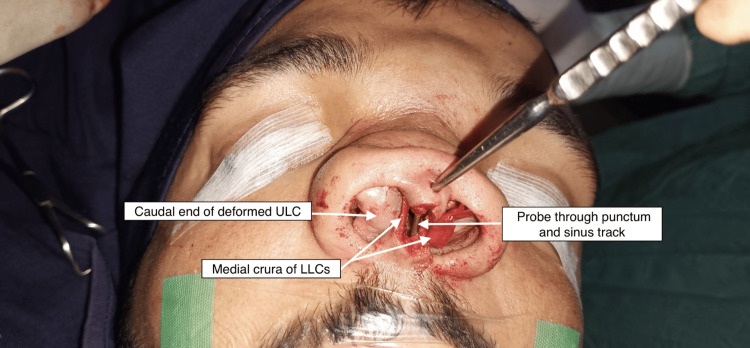
Intraoperative picture of the patient

For these reasons, LA is used almost always in rhinoplasty in addition to general anesthesia (Figures 4, 5). Rhinoplasty was performed with transcollumallar stair step incision with infracartilagenous extensions in vestibule. Dorsal skin flap was raised. During the flap raising, the tract from the collumelar orifice was noted to be extending along the flat depressed dorsum of the nose in the subcutaneous plan to the middle of the broad flattered nasal bones, and there it was connected to the main cyst via a 4 mm opening in the nasal bones. The track was divided and the flap was raised fully to the radix cranially and to the nasal processes of the maxillary bones laterally in the subperiosteal plane. The epithelium of the orifice of track on collumella was burned with the Colorado tip of electrocautery.

The upper lateral cartilages were severely deformed with irregular surfaces and displaced caudally obliterating the internal angle, so they were completely removed preserving the mucosa. As the cyst was occupying a complex anatomical location, it was not possible to remove the cyst in a single piece, but all its components were removed completely. The contents of the cysts were evacuated with a suction cannula (approximately 15 ml of the caseous material). The cyst cavity was lined with an epithelium, which was stripped and curetted, and small remnants were burned with electrocautery. There were only remnants of the cartilageous septum. The bony walls of the cavity were deformed bony septum. All the deformed bony components were removed with the Rongeur and Killian septum gouge (Surgimed Pakistan, Sialkot, Pakistan). The inferior turbinates were in fracture with blunt pressure. After removal of all the bony walls of the cyst, the caseous contents were removed completely from the cyst, and only the septal mucosa was left, which was sewed together with Vicryl 4-0 suture interrupted quilting sutures. A small hole for drainage was made on the right-sided mucosa.

Osteotomies

**Figure 6:**
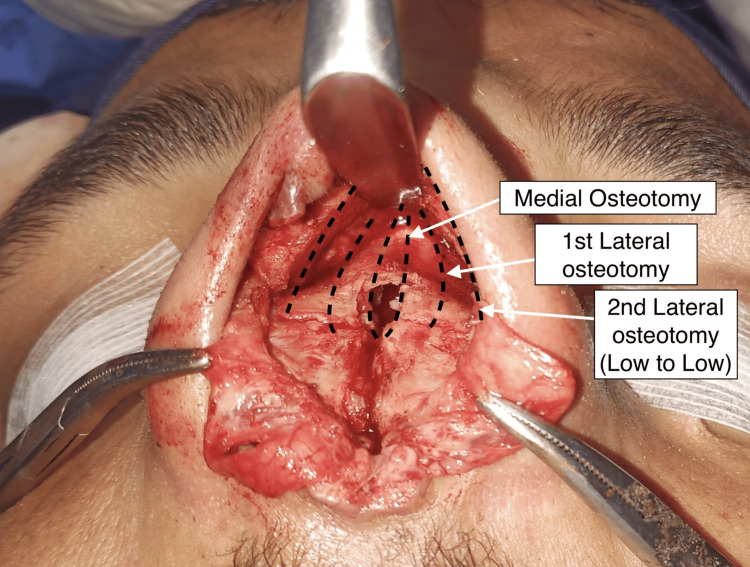
Details of the osteotomies done

Median osteotomy was done with an osteotome extending cranially to the nasal processes of the frontal bones (Figure 6). Transverse osteotomies were done with a lateral 90 degree angled saw at the level of the lateral canthi. The seventh costal cartilage was harvested from the right side, and a strong L strut was contoured. The cranial end of the new L strut was engaged snugly in the cutout made in the nasal processes of the frontal bones, and no screws were used, only the caudal vertical component of the L strut with Prolene 5-0 sutures via holes in the anterior nasal spine, thus achieving a strong nasal nasal support.

As the bony vault was expanded and convex, two lateral osteotomies were done on each side. The first one used a bone-cutting scissors (Leica Surgical, Sialkot, Pakistan)cutting the nasal bone longitudinally and making a flat strip of the nasal bone. The second low-to-low lateral osteotomy was done on the maxillary bone with a lateral cutting saw (Leica Surgical, Sialkoat, Pakistan). The bones were digitally manipulated, and the components aligned at an anatomical position. The cephatic trim of the lower lateral cartilages with turn in flaps, interdomal and transdomal sutures, and medium crural fixation to the L strut was done.

The deformed upper lateral cartilages were carved into flat pieces of the cartilage and placed just causal to the bones with a slight overlap with bones. No suture fixation of the upper lateral cartilages was done. The crushed cartilage graft was fixed as a tip graft, and a diced cartilage graft was placed over the dorsum for minor irregularities. The skin flap was put back, and incisions were closed with a Prolene 6-0 suture for collumellar incision and Vicryl 5-0 for Infracartilagenous incisions. A paraffin gauze for nasal packing was placed in the nostrils. The dorsal splint of plaster of Paris was placed and fixed over the nose.

The total operative time was six hours. Preoperatively, a single dose of 1 g ceftriaxone was given intravenously. Postoperatively, two doses of the same antibiotic was given intravenously and then shifted to oral coamoxiclav for five days. The postoperative period was uneventful. The patient was discharged on the next day. Nasal packing was removed after 48 hours, collumellar stitches were removed on the seventh postoperative day, and the nasal splint was removed at the 14th day.

**Figure 7:**
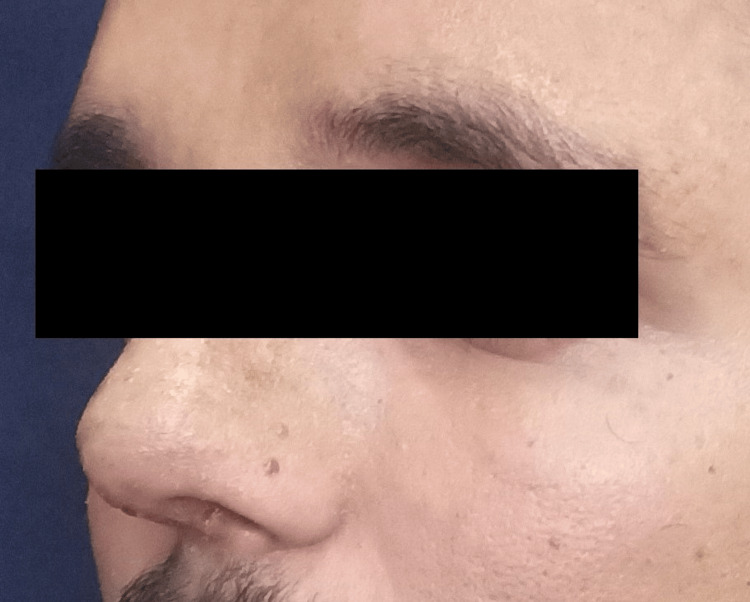
Post-operative pictures of the patient showing good aesthetic results (side view)

**Figure 8:**
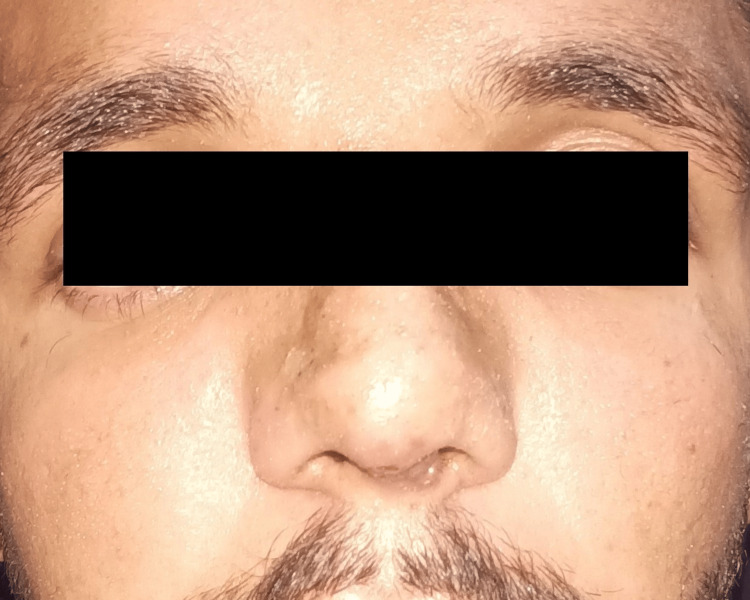
:Post-operative pictures of the patient showing good aesthetic results (front view)

The reconstruction achieved an optimal nasal shape with a good dorsum and tip support, narrower bony vault, good tip projection, and smooth dorsum on lateral view. The patient had no complaints in one month follow-up, and the aesthetic result was satisfying with increased self-confidence as reported by the patient (Figures 7, 8).

Written informed consent for patient information and images to be published was provided by the patient himself, and ethical approval was not required for this individual case study.

## Discussion

The case presented shows a rare nasal dermoid cyst and the treatment course followed to achieve the best results. The present study highlights the new approaches used during surgery that can be replicated by others to treat such cases. NDSCs are the most commonly occuring congenital midline lesions [2,8,9,10]. Bramann published the first report about NDSCs in 1890 [3,4]. The literature shows a number of theories, such as sequestration, trilaminar, and prenasal, to explain the origin of NDSCs. The prenasal theory by Pratt is the most widely accepted one. He described the common embryologic pathway of gliomas, encephaloceles, and naso-frontal dermoid sinus tract [4,7]. The dura is related with dermis in the nasal tip during its extension between the unjoined bones in the skull base to the nasal region. Anomalies occur when the bone tissue is not able to separate the dura from the dermis during the ossification process.

The diagnosis for most of the NDSCs is made during the first three years of life. The diagnosis however can be delayed in some cases until a later age [9]. The same occurred in our case where a 22-year-old adult patient came to get the dermoid cyst surgically removed. The oldest patient that can be tracked down in the literature is a 56-year-old patient with an intracranial extension [10]. There are also some reports about the predominance of male in NSDCs [11,12,13].

In most of the cases, NDSCs are seen as masses in the midline. A sinus opening in the nasal dorsum is typically observed with intermittent secretions and recurrent infections [1,14]. The hair coming out of the opening is found in less than half of the patients [3,11]. Our patient also had no hair coming out of the microhole under the nose. NDSCs are generally seen sporadically, but some cases with family history have also been reported in the literature [1,2]. The case presented in the study had no history of dermoid cyst in the family.

No syndrome is associated with NDSC formation, but they have been associated with other congenital deformities, including cleft defects, aural atresia, or hydrocephalus, in up to 41% of cases [11]. It is important to suspect intracranial extension of lesions for every patient with NDSCs. To rule out or determine the extent of extension, tests like cranial CT and/or MRI are of great importance as they may change the treatment plan opted for the patient [1,2,7]. Bone alterations are clear in cranial CT, which helps in the confirmation of lesions, while MRI clearly shows soft tissue confirming the status of intracranial extension [1,4,7]. The case presented was confirmed to have no intracranial extensions.

Surgical excision is the treatment of choice in NDSCs [1-3,7]. The choice of technique depends on the lesions and the extent of intracranial extension. The most opted technique according to the literature is open rhinoplasty like in our case. It gives a good exposure allowing easy reconstruction of nasal dorsum along with an aesthetically pleasing outcome for the patient [3]. The reasons for choosing this technique are exposure, good aesthetic results, and allowing the reconstruction of the nasal dorsum.

## Conclusions

A nasal dermoid is a rare congenital anomaly that is mostly diagnosed early in life. It requires preoperative evaluation to rule out intracranial extension. Appropriate surgical treatment depends on the location and extent of the lesion. Complete removal of dermoid cysts and sinuses is important to prevent recurrence, which is uncommon and often easily managed. The aim of the given case presentation show how surgical interventions and proper techniques can give good aesthetic results along with the removal of the cyst.
